# Physical Treatment in Heart Disease

**Published:** 1896-02-01

**Authors:** Percy Wilde


### PHYSICAL TREATMENT IN HEART DISEASE.

By Percy Wilde, M.D.

In a recent issue of The Hospital there is an interesting article by Dr. Theodore Fisher on the Schott treatment of heart disease, accompanied by some diagrams of the various physical exercises used by the Nauheim physician in the treatment of this disorder. It is almost unnecessary to state that these are the exercises of Ling, and are familiar to those who have studied the system of Swedish movements. It is not surprising, therefore, that the traditional direction which has been handed down unquestioned from the time of Ling is repeated, viz., that "the tendency of the patient to hold the breath during the movement is to be avoided."

In some papers on "Involuntary Muscular Action," published in the *Edinburgh Medical Journal*, 1880-1, I demonstrated a fact which I never see elsewhere alluded to, that every muscular effort requiring any great exertion is invariably performed at the end of the act of inspiration, the air drawn into the chest being retained during the muscular effort. I explained fully the reason why muscular efforts were performed with less strain under these conditions, and that many muscular efforts would be impossible except they were performed when the lungs were fully expanded with air.

So natural is it to "hold the breath" during any especial exertion that it becomes an involuntary and unconscious act.

As a illustration we may take the condition of a number of men rowing a race. It is only necessary to count the number of strokes rowed in the minute to know the number of respirations taken by each member of the crew. As the arms shoot forward preparatory to making the stroke, each member of the crew is drawing a deep inspiration; as the oars touch the water the inspiration is finished and the breath is held until the oars leave the water again; at the moment when the stroke is completed there is a rapid expiration.

The individual members of the crew are not trained to breathe in this mathematically synchronous manner; they do it involuntarily, because insensible experience has proved that they can obtain the maximum of force with the minimum of strain by holding the breath during the stroke.

The directions, therefore, given by instructors in Swedish movements, that the mouth should be kept open, and holding the breath shall be avoided during the movement, is in direct opposition to nature, and is undesirable, and this is especially the case in the treatment of heart disease.

I described and illustrated in the "Medical Annual," 1891, the exercises I have used for many years in the treatment of diseases of the heart and lungs.

They differ essentially from those employed by Schott. I regard it as absolutely essential that, during the use of direct physical exercise for disease of the heart or lungs, the patient shall be in the horizontal or semi-reclining position. The reason for this is obvious, as otherwise the pressure of the blood in the arteries and veins is disturbed, and there is danger of syncope. When the patient is in the horizontal position, movements designed to directly influence the heart muscle are possible, which would be dangerous if the patient was standing or sitting during the movement.

I also direct that the muscular effort shall be made at the end of an inspiration, the air being retained during the effort. A very little experience will show the practitioner that the lungs and heart are physiologically inseparable. You cannot use physical treatment for the one without stimulating the other, and you cannot cure an ailment of one without both being restored to fuller physiological activity.

A most important point in the treatment of true valvular disease of the heart is, that the symptoms are often due, not to the disease of the valves, but to functional disorders of the nerves of the heart, and of the heart muscle. When there is no dropsy or œdema of the feet, no dyspnœa while at rest, and no evidence of failure of compensation, and this is not uncommon to those affected with valvular disease, it is always wise to remember that functional palpitation, with imperfect respiration, may be the real malady which requires treatment. Imperfect respiration is often the main cause of the dyspnœa in patients with true valvular disease, aad in every case valvular disease of the heart is benefited by securing perfect and complete respiratory movement.

The following exercise will be found to meet the conditions far better than any of those described by Schott, because it acts directly upon the heart and lungs; but being a very potent agent requires to be used with great care: (1) The patient rests in a horizontal position on a couch or bed. The head and shoulders may be slightly raised if necessary. (2) The pulse rate is taken and noted. (3) The operator, standing behind the patient, grasps both the patient's hands, and draws them slowly backwards until they are fully extended behind the head, and the same level as the body. The patient during this movement draws a deep inspiration. (4) The patient is directed to hold the breath and draw the hands downwards, the elbows being on a level with the couch, the movement being continued until the elbows come closely in contact with the chest wall. During this movement the operator resists the movement. (5) As the elbows touch the chest walls the patient makes an expiration, and the operator relaxes the resistance. (6) The patient now makes four or five ordinary respirations, and then the movement is repeated as before. (7) When six movements have been given, with ordinary respirations between, a pause is made while the operator observes the pulse rate, which is usually already slower and stronger. (8) Six more movements are now given in the same way, and the pulse again noted. If the pulse has decidedly improved both in the diminished number of beats with increase in the force and volume, a daily repetition of the movements will give remarkable results within a week. If the movements excite the pulse and embarrass the patient the case is unsuitable for physical treatment. It will surprise most practitioners to find that the latter class of cases are comparatively rare.

